# Supplementary material for: Expression of SIGLEC15 correlates with tumor immune infiltration, molecular subtypes, and breast cancer progression
Source: PLoS One. 2024 Nov 14;19(11):e0313561. doi: 10.1371/journal.pone.0313561 (PMC11563486; doi:10.1371/journal.pone.0313561)
Supplement: S1 Data — (DOCX) [file pone.0313561.s001.docx]

**Title:** Expression of SIGLEC15 correlates with tumor immune infiltration, molecular subtypes, and breast cancer progression

**Huan Lai^1^, Yiyang Liu^1^, Yan Gong^1^, Chuanyu Zong^2^, Wei Zeng^3^*, Honglei Chen^1,2^***

**Table S1.** Patient characteristics in the breast cancer cohorts of TMA.

| **Characteristics** | **Sub-characteristics** | **N (%)** |
| --- | --- | --- |
| Age (years) |  | 45 (27-80) |
| Survival status | Survival  Death | 60 (81.1%)  14 (18.9%) |
| Pathological type | Invasive ductal carcinoma  Invasive lobular carcinoma | 65 (87.8%)  9 (12.2%) |
| Pathological grade | Grade 1  Grade 2  Grade 3 | 14 (18.9%)  49 (66.2%)  11 (14.9%) |
| Tumor size (T) | T1  T2  T3  T4 | 1 (1.4%)  47 (63.5%)  25 (33.8%)  1 (1.4%) |
| Lymph node metastasis (N) | N0  N1  N2 | 46 (62.2%)  24 (32.4%)  4 (5.4%) |
| Distant metastasis (M) | M0  M1 | 74 (100.0%)  0 (0.0%) |
| TNM stage  Intrinsic molecular subtypes | I  II  III  Luminal A  Luminal B  HER2-enriched  Triple-negative | 1 (1.4%)  60(81.1%)  13(17.5%)  15 (20.3%)  31 (41.9%)  14 (18.9%)  14 (18.9%) |
| Total |  | 74 (100.0%) |


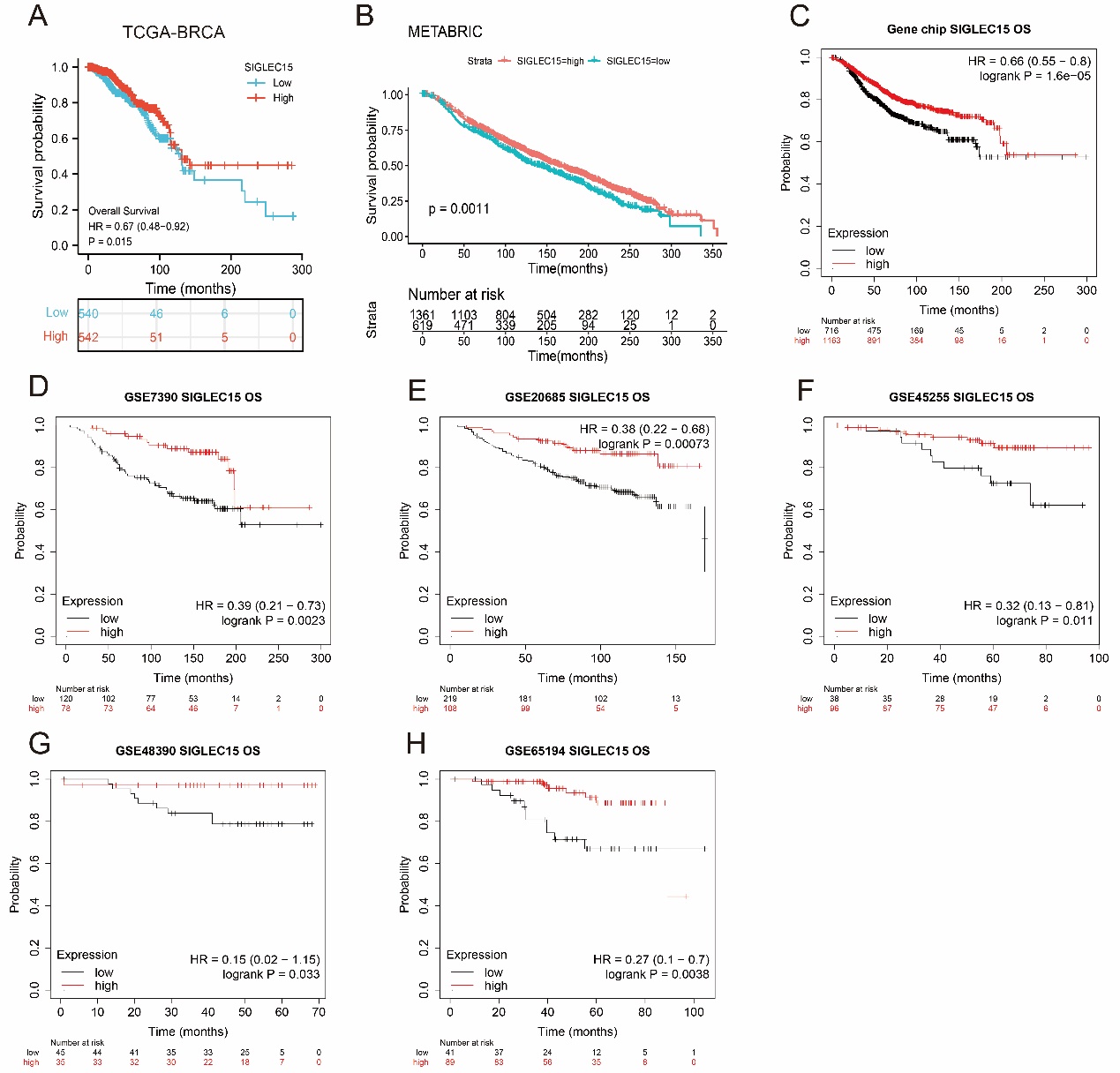


**Figure S1.** OS curves of BRCA according to SIGLEC15 mRNA expression in different databases.

(A)TCGA; (B) METABRIC; (C) Gene chip integrated by KM plotter; (D) GSE7390; (E) GSE20685; (F) GSE45255; (G) GSE48390; (H) GSE65194.


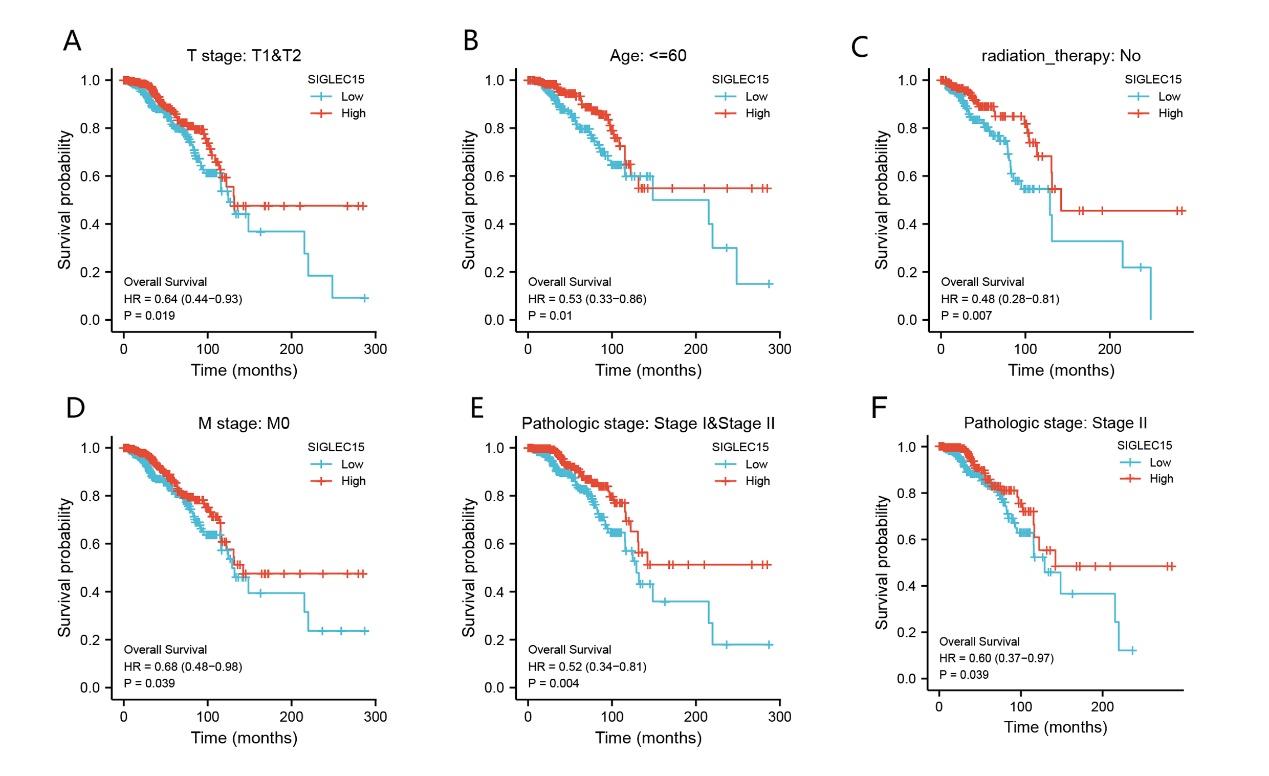


**Figure S2**. OS curves according to SIGLEC15 mRNA expression in different subgroups of BRCA in the TCGA database.

1. T1-T2; (B) Age＜60; (C) radition therapy: No; (D) M0; (E) StageⅠ-Ⅱ; (F) StageⅡ.


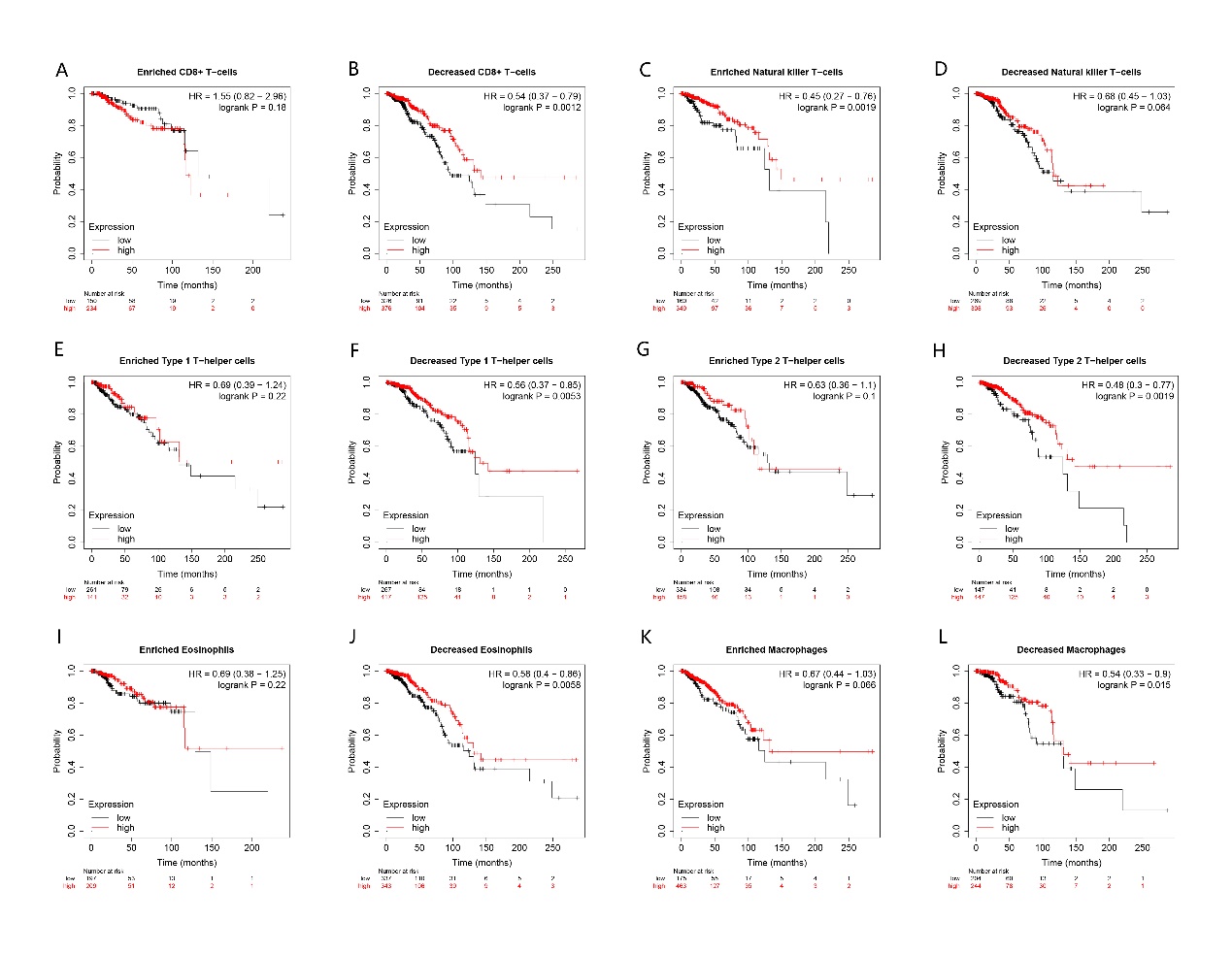


**Figure S3.** OS curves according to SIGLEC15 mRNA in different immune cell subgroups in BRCA plotted by KM plotter.

(A) Enriched CD8^+^ T-cells subgroup; (B) Decreased CD8^+^ T cells subgroup; (C) Enriched Natural Killer T-cells subgroup; (D) Decreased Natural Killer T-cells subgroup; (E) Enriched Type 1 T-helper cells subgroup; (F) Decreased Type 1 T-helper cells subgroup; (G) Enriched Type 2 T-helper cells subgroup; (H) Decreased Type 2 T-helper cells subgroup; (I) Enriched eosinophils subgroup; (J) Decreased eosinophils subgroup; (K) Enriched macrophages subgroup; (L) Decreased Macrophages subgroup.


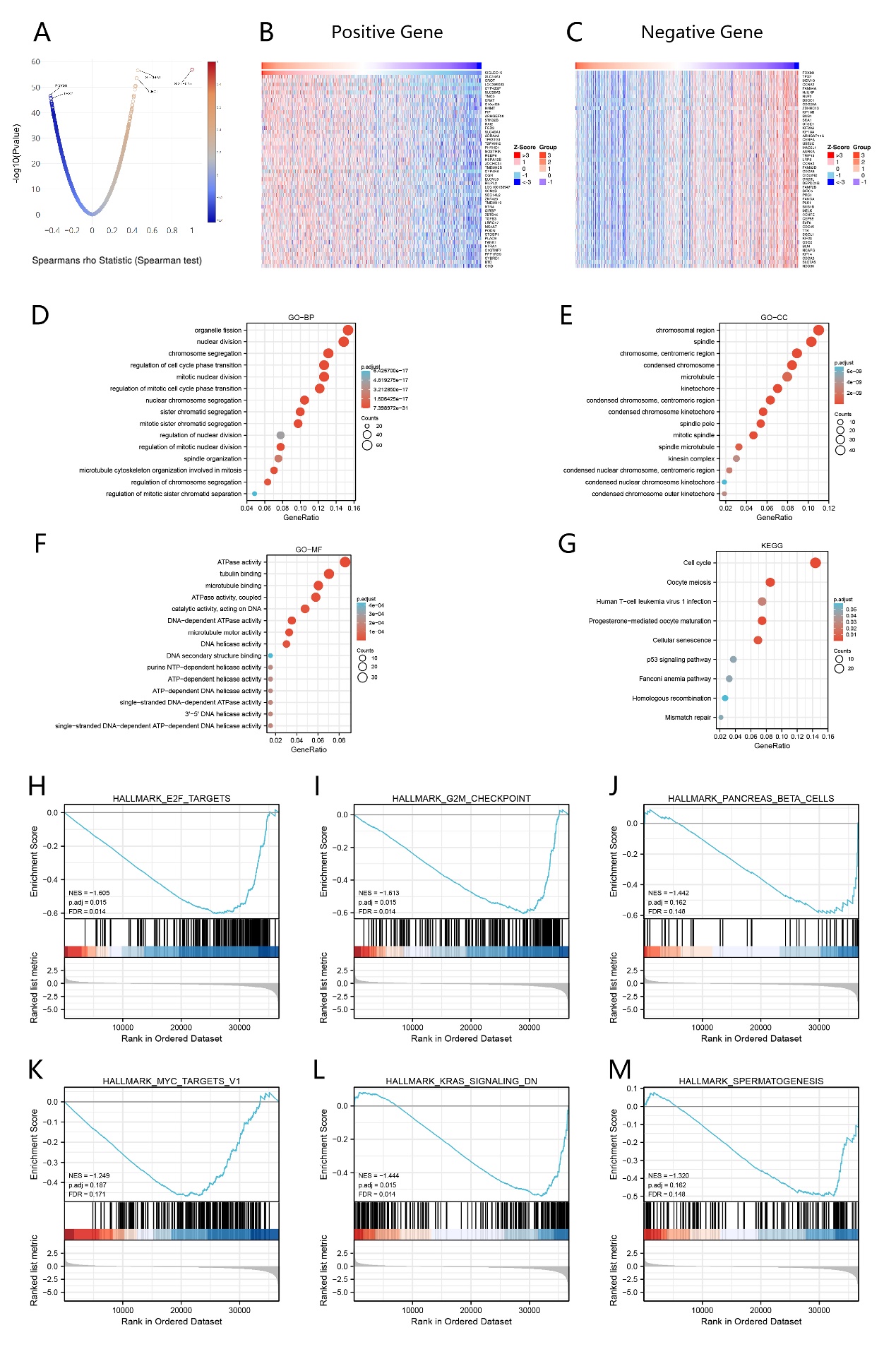


**Figure S4.** Co-expressed genes and functional enrichment analysis of SIGLEC15 mRNA in BRCA.

(A-C) The expression of the top 100 co-expressed genes of SIGLEC15 were examined for association by LinkedOmics; (D-G) GO and KEGG enrichment analysis of co-expressed genes; (H-M) The relevant signal pathways of SIGLEC15 were identified by GSEA software.


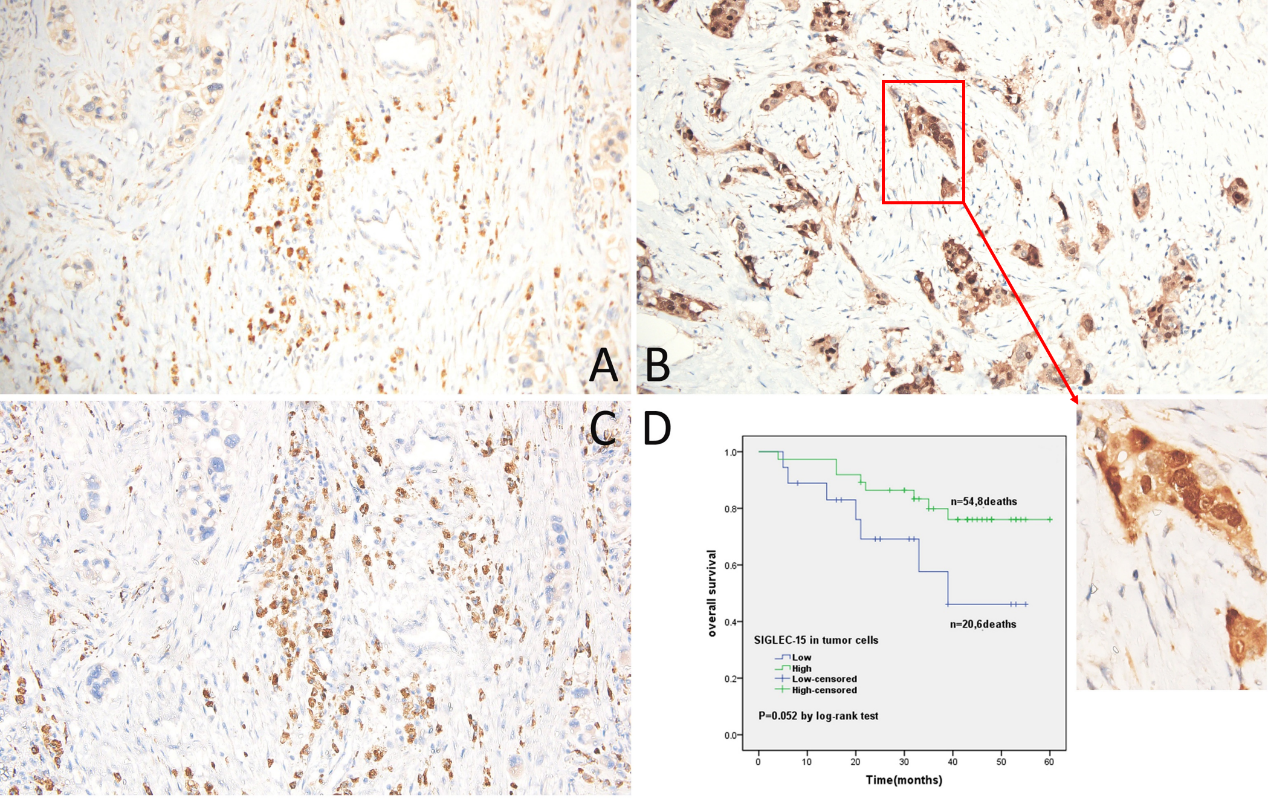


**Figure S5.** Siglec-15 and CD68 protein expression and the prognostic significance of Siglec-15 protein in the BRCA. (A) Siglec-15 protein expression was observed in the cancer cells and macrophages. (B) Siglec-15 protein expression was localized in the cytoplasm and nuclear in the invasive ductal BRCA. (C) CD68 protein expression in the same case with (A), found co-expression of Siglec-15 and CD68.(original magnification A-C ╳200). (D) Low Siglec-15 expression in cancer cells predicted poorer survival and a high mortality rate (P= 0.052) by KM analysis.
