# Supplementary material for: Expression of SIGLEC15 correlates with tumor immune infiltration, molecular subtypes, and breast cancer progression
Source: PLoS One. 2024 Nov 14;19(11):e0313561. doi: 10.1371/journal.pone.0313561 (PMC11563486; doi:10.1371/journal.pone.0313561)
Supplement: Attachment — Submitted filename: Response letter-Chen.docx [file pone.0313561.s002.docx]

**Date: 7/10/2024**

Dear Syrra Mica Hilvano,

Thank you very much for your letter and good advices on our manuscript entitled “Expression of SIGLEC15 correlates with tumor immune infiltration, molecular subtypes, and breast cancer progression”, with the manuscript ID number PONE-D-24-03046. We have revised our review manuscript and will resubmit it. We have carefully studied and addressed the comments raised by the reviewers and journal editors, and have made the corrections using track changes. Point-by-point responses to the reviewer’s comments are listed below this letter.

We hope the revised manuscript is acceptable for publication in your journal. We look forward to hearing from you regarding our submission. And we would be glad to respond to any further questions and comments that you may have.

We would like to express our sincere thanks to the reviewers for the constructive and positive comments.

Reviewer: 1

Comments to the Author

1.While the manuscript presents a number of interesting findings, there are several instances where these results are not fully explained or contextualized and inappropriate words have been used such as what´s more, which could limit the reader's understanding and the study's overall impact. Below are some examples that illustrate this point, though they are not exhaustive:

- The text and figure legends of the manuscript currently do not adequately explain the findings, leading to potential misinterpretation of the data. For instance, the presentation of Figure 3A is currently mislabeled as a heatmap. Additionally, the resolution or format of Figure 3A needs improvement, as the gene labels are currently illegible. Figure 3 A-C appear to represent differential expression analysis and not functional enrichment analysis (Figure D-J are functional enrichment analysis).

Reply: Thank your advices, Figure 3 A-C were co-expressed genes of SIGLEC15. Figure 3A was a volcano plot for correlation analysis of SIGLEC15. And we have improved the resolution of Figure 3A. Figure 3B was a heatmap which shows the top 50 postive co-expressed genes of SIGLEC15. Figure 3C showed the top 50 negative co-expressed genes.

-The organization of subsections within the manuscript currently disrupts the logical flow of the presented data. For instance, the data from Figure 1d is discussed separately from its subsequent analysis related to the clinical parameters of BCa in TCGA, which appears later in Section 3.6. To improve the coherence and progression of the narrative, it is recommended to reorder the sections and join some of the subsections. Moreover, the allocation of data between supplementary and main sections does not appear to be based on the significance or importance of the data.

Reply: Thank your advices, we have restructured the article.

-How is the immune fraction in TCGA datasets analyzed? If tools like CIBERSORT or other deconvolution methods were employed but not explained. Also in section 3.5, the authors do not properly explain the method they used to do the analysis in the body text or the figure legend.

Reply: Thank your advices, we used the ssGSEA algorithm provided in Package R-GSVA to calculate immune infiltration using the markers of 24 immune cells provided in the study by Bindea, et al. (PMID: 24138885). In Section3.5, we used the pROC package [version1.17.0.1] to analyze the data and visualized it using the ggplot2 package [version3.3.3].

-The distinctions between tumor tissues and adjacent normal tissues from TCGA, and normal tissues from GTEx are not clearly made in the text and figure legends.

Reply: Thank your advices, in Fig.1A, the adjacent tissues of TCGA and the normal tissues of GTEX were integrated and labeled as normal tissues, and then the normal and tumor tissues were compared.

-The transition between public datasets to patient data is not clear.

Reply: Thank your advices, we have put public data in front and patient data in the back.

2. A significant overlap between some of the analyses and figures presented in this manuscript and those published in the study by Baihui Li et al. (PMID: 32939323) is noticed. An example is Figure S1 and Figure 1.

Reply: Thank your advices, the study by Baihui Li et al. (PMID: 32939323) simply studies SIGLEC15 in all cancers from TCGA data. These cancers also include breast cancer. Our study focused on breast cancer and relied heavily on TCGA data. Inevitably, there were some overlap in the data. We have changed the S1 to reduce duplicates.

3. In Figures S1 B and C, the analysis shows no significant difference in SIGLEC15 expression between normal and tumor tissues in BCa using TCGA as a reference dataset. Despite the lack of significant differences, the manuscript proceeds to analyze variations between different BCa subtypes in TCGA. The authors should use other commonly used public datasets such as METABRIC, lu et al. PMID: 18297396, Wang et al. PMID: 15721472 to confirm the overexpression of SIGLEC15.

Reply: Thank your advices. Although there was no significant difference in SIGLEC15 expression between normal and tumor tissues in TCGA, a significant difference was observed after incorporating data from GTEx. We believe that the reason for this result might be due to the limited number of normal tissues provided by TCGA, and the fact that the non-tumor tissues in TCGA are derived from adjacent tissue to cancers, which could potentially impact the study's results. In contrast, GTEx data is derived from samples donated by healthy individuals, which are more representative of a healthy population not affected by breast cancer. Additionally, the METABRIC data only includes expression data for tumor tissues and lacks normal tissue data, making it impossible to compare the expression of SIGLEC15. And the METABRIC dataset does not contain clinical classification information for HER2 status, PR status, PAM50 subtypes and TNM stages. Therefore, we did not use the METABRIC study to investigate the expression of SIGLEC15 in breast cancer.

As a supplement, we analyzed the expression of SIGLEC15 in breast cancer and normal tissues after removing adjacent cancerous tissues based on the data from TNMplot, confirming that SIGLEC15 is a high-expression gene in breast cancer. Additionally, by analyzing single-cell databases, we found that SIGLEC15 is primarily expressed in malignant cells and monocytes/macrophages. The results also prove that SIGLEC15 is highly expressed in tumor.

Moreover, in this study, when investigating the expression of SIGLEC15 in different clinical subgroups of breast cancer, normal samples were excluded with the aim of exploring the association between SIGLEC15 and clinical parameters.

4. In Figure 1E-J, the authors have utilized datasets with specific patient characteristics, such as GSE7390 and GSE65194, which provide data on gene expression changes due to therapies. Additionally, GSE20685 offers insights into environmental or lifestyle factors, like smoking, that may impact BCa at the molecular level. GSE4525 explores the genetic differences between hereditary and sporadic breast cancer cases. However, the manuscript does not discuss these distinctive characteristics when presenting the data, nor does it address their impact on survival outcomes between the low and high SIGLEC15 expression groups. These specific factors could significantly influence the survival rates observed. For instance, the effects of therapy might explain better survival, even when the overexpressed gene contributes to tumor progression. The authors should use other commonly used public datasets such as METABRIC, lu et al. PMID: 18297396, Wang et al. PMID: 15721472 to confirm the OS.

Reply: Thank your advices. Upon reviewing the original texts, we found that GSE7390 studies untreated lymph node-negative breast cancer patients, while GSE65194 indeed comprises breast cancer samples that have undergone radiotherapy and chemotherapy. We have emphasized this in the main text. The original article for GSE20685 does not mention environmental factors affecting breast cancer; it primarily discusses the correlation between six molecular subtypes of breast cancer and clinical outcomes. GSE45255 investigates the effects of immunological, proliferative, and molecular subtypes on the prognosis of breast cancer and does not mention hereditary and sporadic breast cancer, so we believe that the data from GSE45255 is representative. Our research primarily aims to examine the impact of SIGLEC15 expression levels on overall survival (OS), so I consider the above datasets suitable for validating our conclusions. Additionally, we analyzed the impact of SIGLEC15 expression on OS using METABRIC data, which also validated our conclusions.

5. Given that the majority of analyses in this manuscript utilize publicly available datasets primarily at the mRNA level, such as TCGA, it is important to consider extending these analyses to include protein expression data. Protein differential analysis could be particularly insightful, as protein expression does not always correlate directly with mRNA levels due to post-transcriptional and post-translational modifications.

Reply: Thank your advices. Our research is indeed primarily based on mRNA. Since SIGLEC15 is a relatively new gene, we have not found any other public protein databases containing Siglec-15 protein. Moreover, we have validated the presence of Siglec-15 in tumor tissues at the protein level through immunohistochemistry and mIHC experiments.

6. In Figure 4, the IHC sections illustrating SIGLEC-15 expression are currently not presented with sufficient clarity. Each section that demonstrates the expression of SIGLEC-15 should be paired with a corresponding negative control section to clearly establish baseline staining. Additionally, the transitions between ductal in situ carcinoma and ductal invasive carcinoma is not distinctly marked in the current presentation. Moreover, macrophages in tumor stroma have distinct markers that help in their identification. Without the use of these specific markers, distinguishing stromal macrophages from other cell types in the stroma based solely on morphology in IHC is inaccurate. The authors address this in Figure 5, but it can not be concluded from figure 4. Additionally, in Figure 5, the authors have not provided explanations regarding the cellular identities associated with the CK, CD68, or CD8 markers.

Reply: Thank your advices. Every IHC experiment also designed negative control, almost negative expression of Siglec-15 in the normal breast epithelium. Our TMA block included 75 breast cancer tissues and corresponding non-cancerous tissues. We updated the Fig.S5 including Siglec-5 and CD68 images in the same section detected by IHC. In the Fig.5, we just want to confirm co-expression of Siglec-15 protein with macrophages and T cells. We didn’t detect the TMA samples using mIHC.

7. The study utilizes tissue microarray samples from 75 breast cancer patients to evaluate Siglec-15 protein expression, with histological examples provided. Although Siglec-15 protein levels are presented in Table 1, this association is not explicitly mentioned in the text with refferng to the corresponding data. Additionally, while patient samples are integral to the findings, their significance has not been adequately emphasized in both the introduction and discussion sections. Moreover, mRNA expression levels of Siglec-15 in the same patients have not been showcased. To improve data presentation, I recommend depicting both protein and mRNA expression levels in separate bar or dot graphs.

Reply: Thank your advices. We have revised them.

Reviewer: 2

Comments to the Author

1. As a general comment for the document, the authors should standardize where they are using “SIGLEC15” and “Siglec-15” as it is not consistent in the document. From my understanding SIGLEC15 is the gene whereas Signlec-15 is the protein. If that's the case, please italicize the gene name so it's clear without any confusion.

Reply: Thank your advices. We have revised them.

2. Section 3.5 discusses the diagnostic significance of SIGLEC15 for molecular subtypes but has not been explained in detail. It would be beneficial to add some more context to this in the discussion to understand the significance of these findings.

Reply: Thank your advices. We have revised them.

3. Abbreviations should be kept consistent (for example, KM used for kaplan Meier curve used be used consistently in the document).

Reply: Thank your advices. We have revised them.

4. In section 3.11, were there any significant correlations found between Siglec-15 expression and the immune cell abundance/levels other than the co-expression? (Something similar to what has been done in section 3.12 with chi-square analysis.)

Reply: Thank your advices. We have revised them.

From above all, so we believe this manuscript may be of particular interest to the readers of your journal.

Best regards,

Hong-Lei Chen, MD., Ph.D.

Department of Pathology

Zhongnan Hospital of Wuhan University

185 Donghu Road, Wuchang District

Wuhan, Hubei 430071

Office: 86-27-67811732

Email: hl-chen@whu.edu.cn
