# Supplementary material for: Expression of SIGLEC15 correlates with tumor immune infiltration, molecular subtypes, and breast cancer progression
Source: PLoS One. 2024 Nov 14;19(11):e0313561. doi: 10.1371/journal.pone.0313561 (PMC11563486; doi:10.1371/journal.pone.0313561)
Supplement: Attachment — Submitted filename: Response letter-Ch.docx [file pone.0313561.s003.docx]

**Date: 10/12/2024**

Dear Amy McCart Reed,

Thank you very much for your letter and good advice on our manuscript, “Expression of SIGLEC15 correlates with tumor immune infiltration, molecular subtypes, and breast cancer progression,” with the manuscript ID number PONE-D-24-03046R1. We have carefully revised our manuscript, responded to each point raised by the academic editor and reviewer(s), and will resubmit it.

We hope the revised manuscript is acceptable for publication in your journal. We look forward to hearing from you regarding our submission. Moreover, we would be glad to respond to any further questions and comments that you raised.

We would like to express our sincere thanks to the academic editor and reviewers for their constructive and positive comments.

Comments to the Author

I have reviewed in detail and find some additional areas that need addressing. Your manuscript would be greatly improved with English language editing. Please can you explain the need to add an additional author?

Reply: Medjaden Inc. have supported its assistance in the preparation of our manuscript, if you think our manuscript need to be polished languages again, we agree with your advice.

In the response to reviewers you say you didn’t use METABRIC, but it is in both the methods and the supplement?

Reply: Sure, you are right, we used METABRIC, which is an important database to investigate breast cancer bioinformatics.

Very unconventional statement to include in the introduction – please remove- Its role in cancer immune regulation was first characterized in 2019 by Prof. Chen [15].

Reply: Thank your advice, we changed the statement.

How many cases did you do mIHC on? Was it also the TMA? Where is that data (to complement the images in Fig5) – do you have some co-expression plots?

Reply: Ten cases of FFPE breast cancer blocks were collected from Department of Pathology, Zhongnan Hosipital of Wuhan University. We just wanted to confirm co-expression of Siglec-15 with CK, CD68, and CD8, respectively.

Is there sufficient tumour associated normal in the TMA to support your conclusions about normal expression? A TMA typically focusses on a tumour rich region - an H&E would be helpful.

Reply: We used TMA slides comprising 75 breast cancer tissues and corresponding non-cancerous tissues, we updated the figures and added the H&E images(Figures listed below).

Table S1 – which datasets?

Reply: TMA data, we added the messages.

Table 2 – the first column is very confusing – it is not clear which variables are being analyzed.

Reply: We revised the column, to let the reader understand easily.

Figure S5 needs more annotation and a higher powered view to support the subcellular localization statements.

Reply: We updated the higher power images.

In the discussion you mentioned SIGLEC mRNA is prognostic in TCGA/METABRIC but you don’t discuss that is it not an independent prognostic marker as shown by your Cox multivariate analysis.

Remove the phrase ‘prognostic boon’ from the discussion.

Reply: we changed the phrase.

This sentence – ‘Elevated SIGLEC15 expression is indicative of a potential Luminal subtype within the sample’ is not evidenced by your study. Please remove it.

Reply: Thank your advice, you read carefully it. We removed this sentence.

From above all, so we believe this manuscript may be of particular interest to the readers of your journal.

Best regards,

Hong-Lei Chen, MD., Ph.D.

Department of Pathology

Zhongnan Hospital of Wuhan University

185 Donghu Road, Wuchang District

Wuhan, Hubei 430071

Office: 86-27-67811732

Email: hl-chen@whu.edu.cn
